# Detection of breast cancer using machine learning and explainable artificial intelligence

**DOI:** 10.1038/s41598-025-12644-w

**Published:** 2025-07-24

**Authors:** Tharunya Arravalli, Krishnaraj Chadaga, H Muralikrishna, Niranjana Sampathila, D. Cenitta, Rajagopala Chadaga, K. S. Swathi

**Affiliations:** 1 Department of Electronics and Communication Engineering, Manipal Institute of Technology, Manipal Academy of Higher Education, Manipal, Karnataka 576104 India; 2 Manipal Institute of Technology, Manipal Academy of Higher Education, Manipal, 576104 India; 3 Department of Biomedical Engineering, Manipal Institute of Technology, Manipal Academy of Higher Education, Manipal, 576104 India; 4 Department of Mechanical and Industrial Engineering, Manipal Institute of Technology, Manipal Academy of Higher Education, Manipal, 576104 India; 5 Department of Health Care and Hospital Management, Prasanna School of Public Health, Manipal Academy of Higher Education, Manipal, 576104 India

**Keywords:** Breast cancer, Machine learning, Explainable artificial intelligence, Diagnosis, Ensemble classifier, Attribute selection, AI in healthcare, Cancer, Health care

## Abstract

Breast cancer is characterized by the proliferation of abnormal breast cells that eventually turn into malignant tumors. These cancer cells can metastasize to be life-threatening and fatal. An intricate mix of environmental factors and individual genetic composition can lead to the formation of this deadly carcinoma. Improvements in the diagnosis and treatment of cancer are essential given the rising incidence of breast cancer. Over the past few decades, machine learning has helped provide accurate medical diagnosis results. Therefore, this study used diagnostic characteristics of patients and multiple machine learning classifiers to identify breast cancer. Incorporating explainable artificial intelligence techniques revealed the underlying factors for the model predictions, adding a layer of transparency and interpretability. Out of the algorithms, random forest showed the best result, an F1-score of 84%. The stacked ensemble model, which combines the strengths of different models, obtained an F1-score performance of 83%. The research emphasized the results obtained by explainers such as SHAP (SHapley Additive exPlanations), LIME (Local Interpretable Model-agnostic Explanations), ELI5 (Explain Like I’m Five), Anchor and QLattice (Quantum Lattice) to decipher the findings. Interpretable algorithms can be applied in the medical sector to assist practitioners in predicting breast cancer, reducing diagnostic errors, and improving clinical decision-making.

## Introduction

Breast cancer is a condition where tumorous breast cells proliferate uncontrollably^1^. After skin, breast cancer is the most common cancer detected in women. In 2022, approximately 2.3 million women across the globe were diagnosed with breast cancer, and the disease claimed the lives of 670,000 of them^2^. Although women are more likely than men to have breast cancer, this does not imply that men will not develop this dangerous disease^3^. Based on the site of cancer initiation in the breast there are different types of breast cancer (invasive and non-invasive breast cancer)^3^. The primary triggers for this illness are aging, a family history of cancer, genetic alterations inherited through heredity, hormonal changes, way of life, and environmental variables^2^. If this condition is left untreated, it causes the disease to spread to other organs, which leads to multi-organ failure and death^4^. Research has shown that early cancer detection can increase survival rates^1–3^. The symptoms in immediate need of attention are a lump swelling in one’s breast, chest, or armpit, nipple discharge containing blood, breast pain and change in the breast size^5^.

**Fig. 1:**
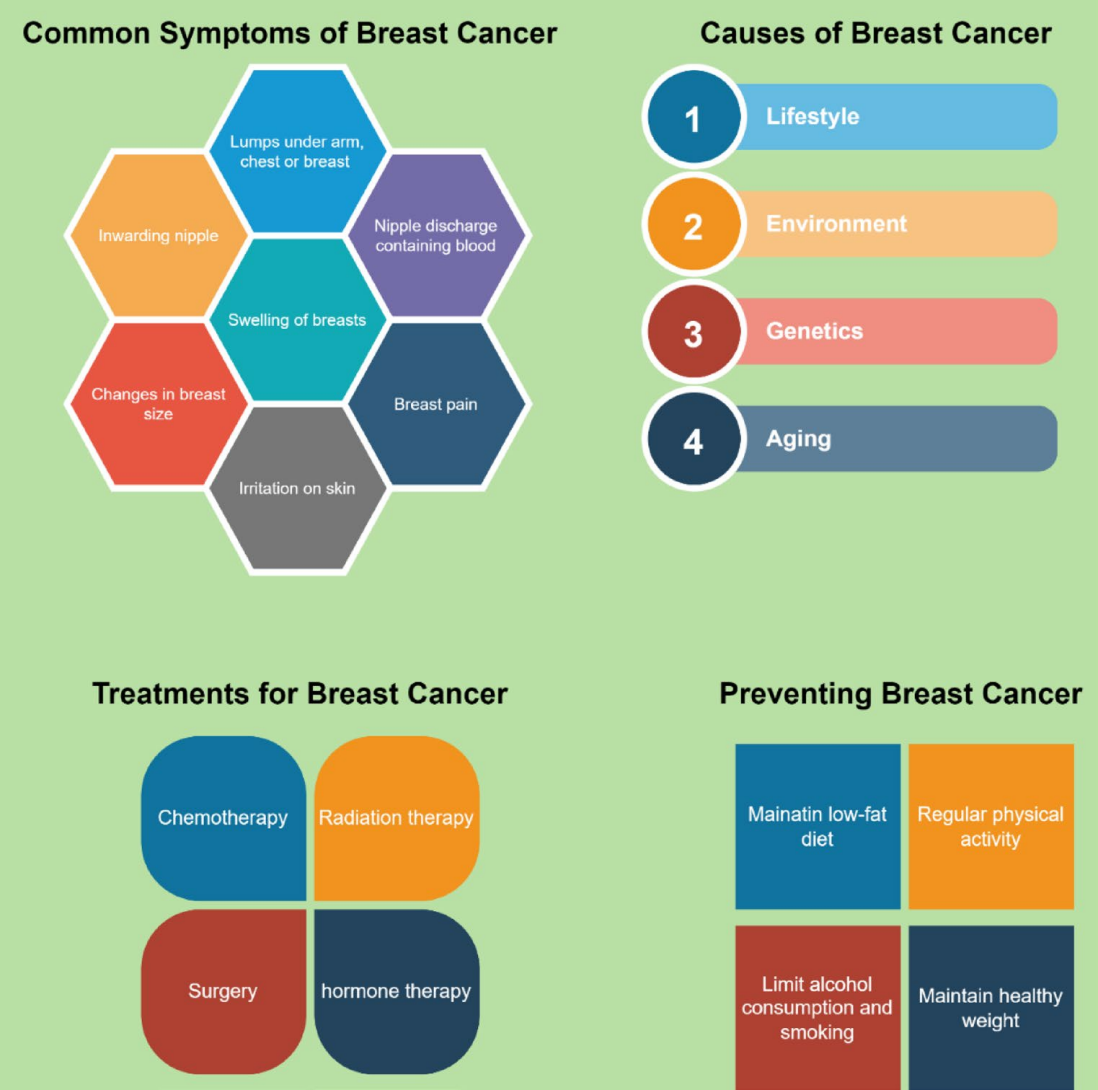
Common symptoms, causes, treatments, and methods for breast cancer.

A mammography is a breast cancer screening test that reveals breast density, calcification, various abnormalities, and a mass (breast lump)^6^. A few other tests for detailed diagnosis include MRI (magnetic resonance imaging), progesterone receptor test, PET (positron emission tomography) scan, gene test, biopsy, estrogen receptor test, and CT (computed tomography) scan. The treatments for breast cancer are surgery, radiation therapy, chemotherapy, hormone therapy, targeted therapy, and immunotherapy^4^. Even though genetics cannot be altered, healthy lifestyle adaptations can reduce the risk of breast cancer^7^. Limiting alcohol and smoking, maintaining a healthy weight, regular exercise, breastfeeding in case of a baby, and avoiding hormone therapy after menopause can be considered as factors to reduce breast cancer. Figure 1 shows an overview of breast cancer.

Some drawbacks of current diagnosis techniques include limited imaging capabilities, overlooking symptoms, expensive procedures, and slow processing speeds^8^. According to the National Institutes of Health, human error (96.3%) is the major source of diagnostic adverse outcomes. Integrating ML in the diagnosis can help overcome this. The ability of computers to learn from data has proven to be quite beneficial in predicting various diseases. Studies have shown that machine learning (ML) can be used to assist clinicians in diagnosing breast cancer^9^. This research focuses on identifying a machine learning-based diagnostic method to predict breast cancer diagnosis. ML can analyze medical data, assist medical professionals, minimize human errors, and result in expedited diagnoses and treatment plans^9^. Explainable Artificial Intelligence (XAI) is a set of protocols and methods that allow machine learning algorithms to generate human-understandable results. XAI can validate model results, enhance the stability and predictability of interpretable models and creates opportunities for error detection and correction^10^.

Various studies have been conducted to evaluate machine learning’s use in medical prospects. Sharma et al.^11^ conducted a study using the WDBC dataset containing 569 instances with 32 attributes. It focused on comparing 3 different machine learning techniques for breast cancer detection. Out of these, kNN gave the best result with an accuracy of 95.90%, indicating robustness in detection. A similar study using the same dataset was done by Thilaka et al.^12^ which explicitly chose five major features for training the model. SVC was the best-performing model, with a superior accuracy of 93%. Silva et al.^13^ used a mendeley dataset for detecting breast cancer in Indonesian women. It contained 400 patient cases, out of which 200 were diagnosed with breast cancer. The XGBoost model gave the highest accuracy of 85%. SHAP and CRISP-ML(Q) were used for explainability and quality assurance of ML outputs. In Munshi et al.^14^, an ensemble model was employed, consisting of a support vector machine (SVM) and random forest (RF) model. It performed meticulously with an accuracy of 99.99%. Islam et al.^15^ designed a predictive model for classifying breast cancer on a dataset of 500 patients. This data was collected from Dhaka Medical College Hospital, Bangladesh. Five ML algorithms were implemented, and Xgboost performed well with an accuracy of 97%. SHAP (Shapley Additive explanations) was applied to the model to ensure transparency and trust in the results. Oztekin et al.^9^ compared their XAI model with radiologist reviews to identify breast cancer. In this study, a novel breast cancer dataset was constructed using 752 different patient’s benign and malignant lesions as diagnostic labels. They used six classifiers and XGBoost gave the best results with 94% accuracy. The performance of the AI model was compared with a radiologist’s diagnosis (83.95% accuracy) concluding machine learning algorithms are reliable. The results were further interpreted by SHAP and LIME (Local Interpretable Model Agnostic Explanations) XAI techniques to identify critical features. Imouokhome et al.^16^ classified breast tumors as benign or malignant on histopathological images using a pre-trained ResNet50 model. The accuracy obtained was 96.84%. The dataset was obtained from the public repository Kaggle. It contained 7909 images of breast tumors, collected from 82 patients by Surgical open biopsy method. This study used advanced XAI techniques for deep learning interpretation. A detailed analysis of the articles mentioned above is presented in Table 1.

**Table 1:** Articles that use ML for the diagnosis of breast cancer.

S.No.	Author	Dataset	Best ML model	XAI techniques	Accuracy
1.	Sharma et al.^11^	Wisconsin Diagnosis Breast Cancer dataset	kNN	None	95.90%
2.	Thilaka et al.^12^	WBCD dataset	SVC	None	93%
3.	Silva et al.^13^	Mendeley data	XGBoost	SHAP	85%
4.	Munshi et al.^14^	Breast Cancer Wisconsin dataset	(RF + SVM) ensemble model	SHAP	99.99%
5.	Islam et al.^15^	Patient data from Dhaka Medical College Hospital	XGBoost	SHAP	97%
6.	Oztekin et al.^9^	Newly created Breast-XD dataset	XGBoost	SHAP LIME	94.43%
7.	Imouokhome et al.^16^	BreakHis dataset from Kaggle Challenge	ResNet50	Integrated Gradient, GradientShap And Occlusion	96.84%

We found that most studies have not used multiple heterogeneous XAI techniques for interpretation. Further, important markers identified were not validated using other techniques. To solve these issues, the current research includes the following additional contributions:

- The dependency of various features on diagnosis is identified by utilizing mutual information. The information gain obtained can be utilized to understand the importance of the marker for breast cancer diagnosis.
- A customized ensemble classifier is created in this research using the stacking methodology. The results obtained by this algorithm are more reliable since it considers all the techniques during prediction.
- Five XAI techniques are used to identify the feature contribution: LIME, Anchor, SHAP, QLattice, and Eli5. The important markers analyzed are further validated using mutual information and inferential statistics.

Major limitations observed in the study are limited dataset size and inconsistencies of feature selection with other research.

## Materials and methods

### Dataset description

This paper used the “UCTH Breast Cancer Dataset” for machine learning analysis. The patient data was uploaded to a reliable repository called Mendeley Data in the year 2023^17^. It was obtained from the University of Calabar Teaching Hospital, Nigeria, by observing 213 patients over two years. It contained nine features: age, menopause, tumor size, involved nodes, area of breast affected, metastasis, quadrant affected, previous history of cancer, and diagnosis result. Age and tumor size are continuous variables. Menopause, involved nodes, breast, metastasis, breast quadrant, and history are categorical variables. The categorical target variable is ‘diagnosis result’, which contains ‘0’ for benign and ‘1’ for malignant diagnosis. Table 2 presents a comprehensive description of the features within the dataset.

**Table 2:** Explanation of the dataset’s features.

S.No	Feature name	Description
1.	Age	The patient’s age at the time of diagnosis.
2.	Menopause	The menopausal status of the patient.
3.	Tumor size	The size of the excised tumor, measured in centimeters.
4.	Involved nodes	The number of axillary lymph nodes affected by tumor spread.
5.	Breast	Whether the tumor is located on the left or right side.
6.	Metastasis	Whether the cancer has metastasized to other parts of the body or organs.
7.	Breast quadrant	The breast is divided into four sections, with the nipple at the center, indicating the tumor’s precise location.
8.	History	Whether the patient has a personal or family history of cancer.
9.	Diagnosis result	Classifying the tumor into benign and malignant.

### Statistical preprocessing

**Fig. 2:**
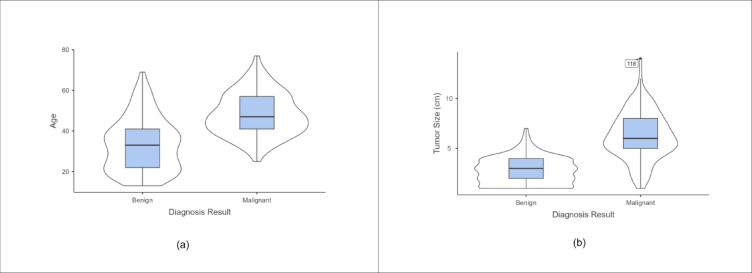
Violin plots. (**a**) Age (**b**) Tumor size.

The research utilized Jamovi to draw statistical and descriptive conclusions^18^. The descriptive analysis for continuous variables is given in Table 3. For visualizing the distribution of numerical data, violin plots are shown in Fig. 2. According to the plot, older women are more prone to malignant breast tumors than younger women. Greater tumor size indicated a malignant diagnosis. T-tests were utilized to check the importance of the continuous features. The feature is considered significant if the p-value is less than 0.001. From Table 4 it is concluded that both tumor size and age are necessary features.

**Table 3:** Descriptive analysis of continuous variables.

	Diagnosis result	*N*	Missing	Mean	Median	SD	Range	Minimum	Maximum
Tumor size (cm)	Benign	120	0	2.72	3	1.34	6	1	7
	Malignant	92	1	6.29	6	2.38	13	1	14
Age	Benign	120	0	33.21	33	13.04	56	13	69
	Malignant	93	0	48.27	47	10.44	52	25	77

**Table 4:** Independent samples t-test.

		Statistic	df	*p*
Age	Student’s t	-9.11	211	< 0.001
Tumor Size (cm)	Student’s t	-13.85	210	< 0.001

Inference: Age and tumor size are significant features

**Fig. 3:**
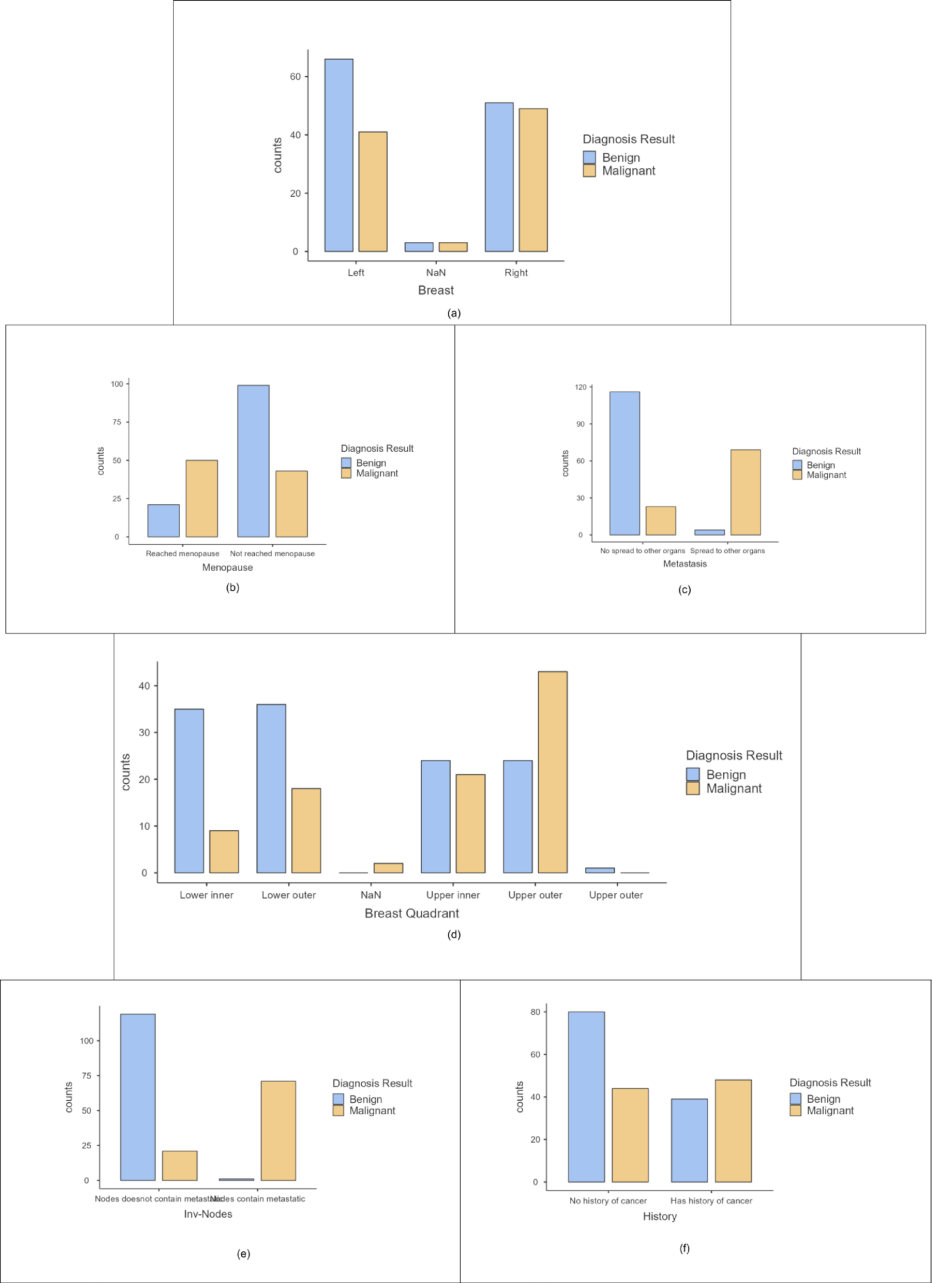
Bar plots for categorical variables (**a**) Breast (**b**) Menopause (**c**) Metastasis (**d**) Breast quadrant (**e**) Involved nodes (**f**) History.

Categorical variables are analyzed using bar plots shown in Fig. 3. It showed the number of patients with benign and malignant tumors for different features. From the graphs, it is interpreted that breast cancer is not adverse in patients who haven’t reached menopause. The diagnosis of this cancer is also observed when the tumor has spread to the auxiliary nodes. Metastasis is noted to be prominent in the case of breast cancer. Malignant tumours have been reported when it affected the upper outer quadrant. Patients with a previous history of cancer are more prone to be diagnosed with this carcinoma. These bar plots help analyze the dataset in-depth. A chi-square test is done to identify significant categorical features. The results are shown in Table 5. The features: Menopause, Involved nodes, Breast Quadrant, and Metastasis are inferred to be necessary attributes as per chi-square tests.

**Table 5:** Chi-square test.

S.No	Attribute	*p* value
1	Menopause	< 0.001
2	Involved nodes	< 0.001
3	Breast	0.287
4	Breast quadrant	< 0.001
5	Metastasis	< 0.001
6	History	0.005

Inference: Menopause, Involved nodes, Breast Quadrant, and Metastasis are significant features.

### Data preprocessing

Data preprocessing enables converting unprocessed data into a format that is easy to read and use for analysis. This research used preprocessing to avoid using missing values as well as outliers and to optimize the input feature size during analysis. Data shuffling was first done to prevent the model from recalling the order. The dataset had 13 null values, represented as ‘NaN’, that are later removed to attain uniformity. Label encoding was used to convert categorical text data into numerical values since machine learning algorithms need numerical input. It assigns a distinct integer to each category that machine learning algorithms can process. Data scaling avoids any bias towards the larger values in the dataset. Max-Abs-scaling was used to transform all the numbers between 1 and − 1 using its maximum absolute value.

**Fig. 4:**
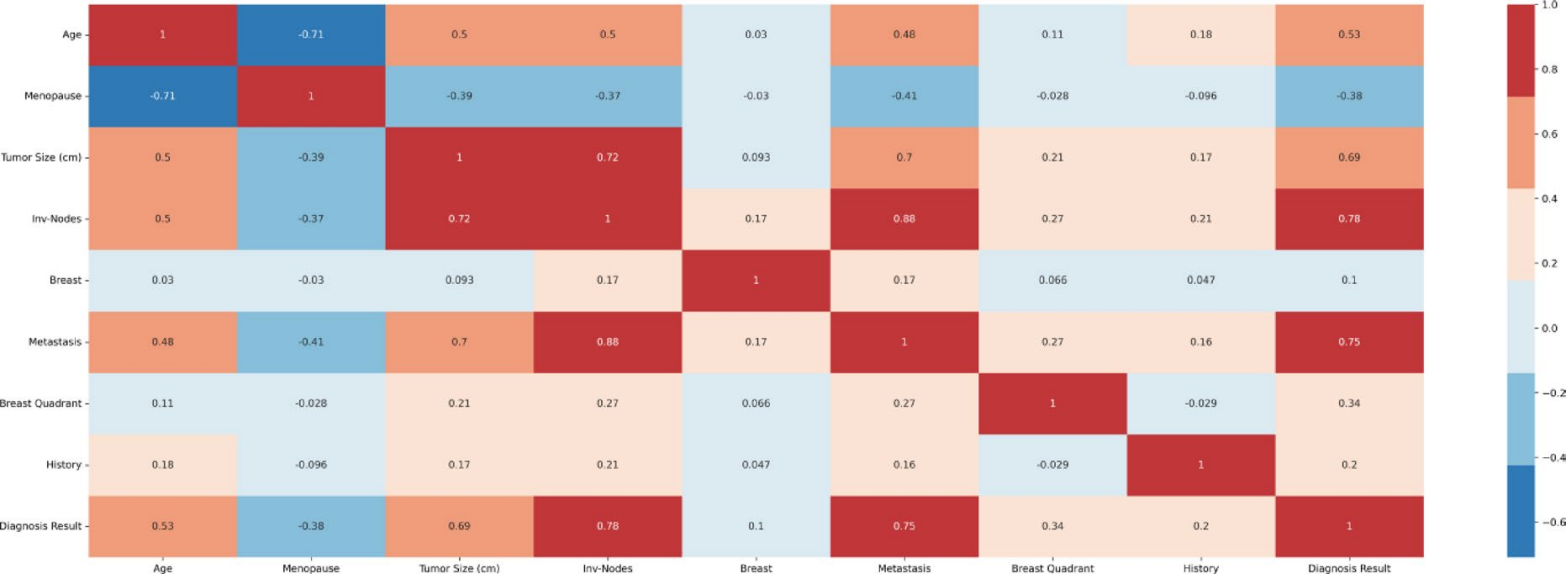
Pearsons correlation heatmap.

**Fig. 5:**
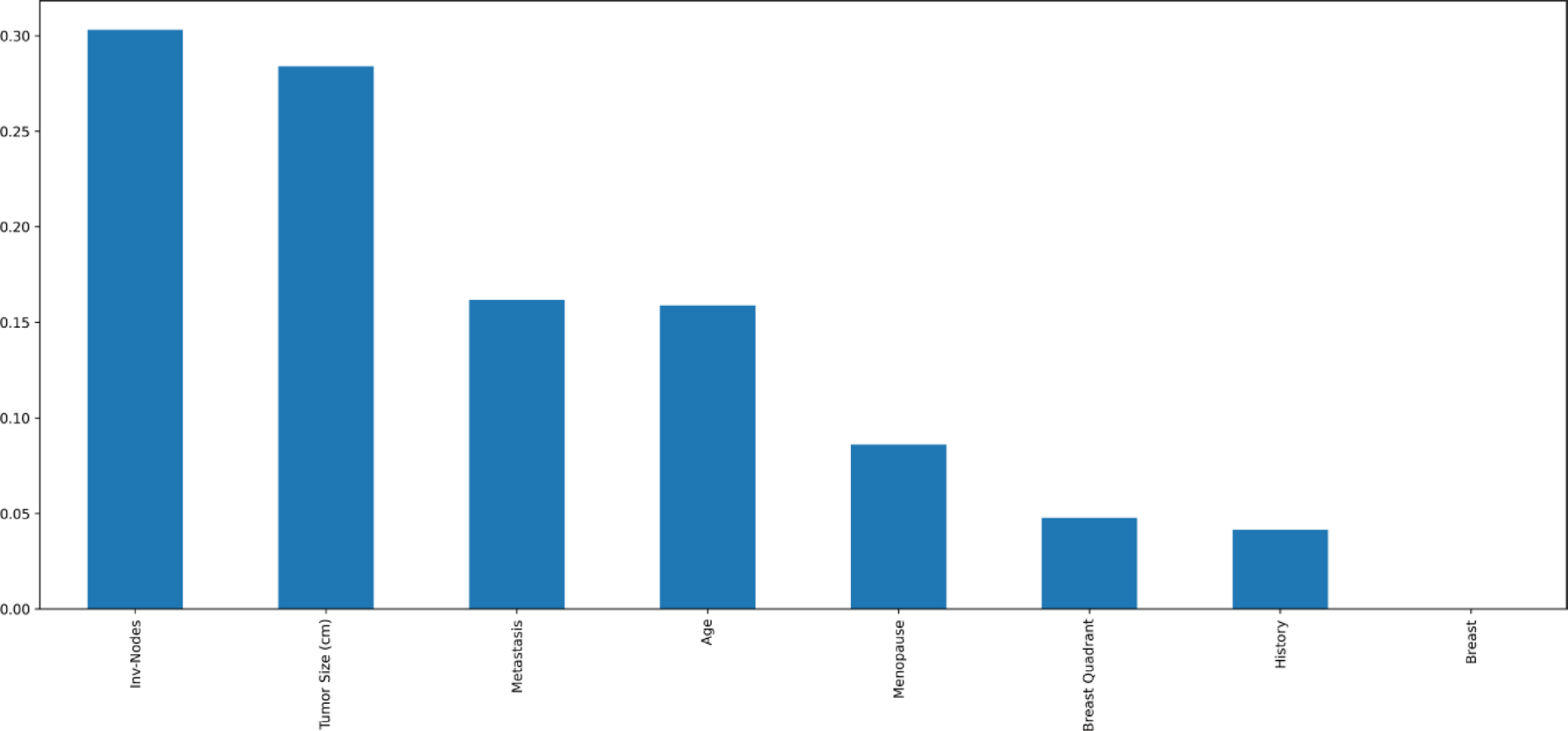
Mutual information of features.

**Fig. 6:**
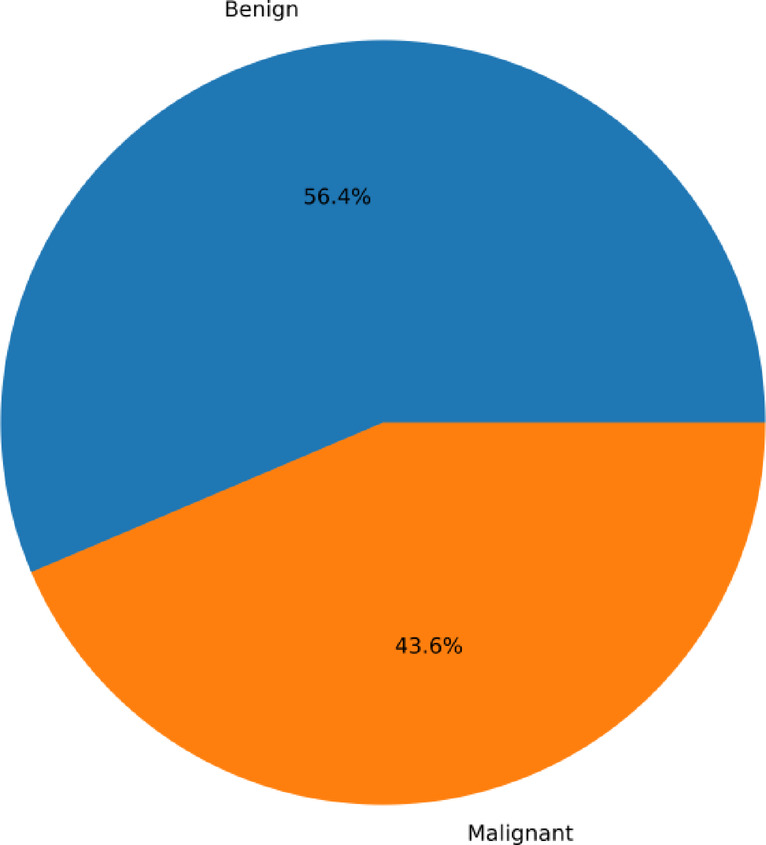
Diagnosis cohort.

Mutual information and Pearson’s correlation are utilized to determine the important features. The correlation coefficients between any two sets of characteristics are displayed as a heatmap in Pearson’s correlation matrix. The values 1,0 and − 1 indicate positive, zero, and negative correlation, respectively. The heatmap is shown in Fig. 4, according to which Involved nodes, Metastasis, Tumor size, and Age were highly correlated to the diagnosis result. Mutual Information is a univariate filtering method where the significance of a feature is calculated separately. It shows the dependency between two variables with the concept of entropy. In Fig. 5, the qualities are ordered in order of importance. The important features, according to mutual information, are involved nodes, tumor size, metastasis, age, menopause, breast quadrant, and history. The visualization for the distribution of the target variable (diagnostic result) is shown as a pie chart in Fig. 6. From the figure, it is seen that there is a slight imbalance in the data, which might induce biases in the model performance. The Borderline-SMOTE is applied to the training data to balance the classes by creating synthetic samples. This balanced the dataset to 50% for both cases^19^. Furthermore, the dataset was divided into test and training data in the proportion of 30:70.

### Machine learning and explainable artificial intelligence

**Fig. 7:**
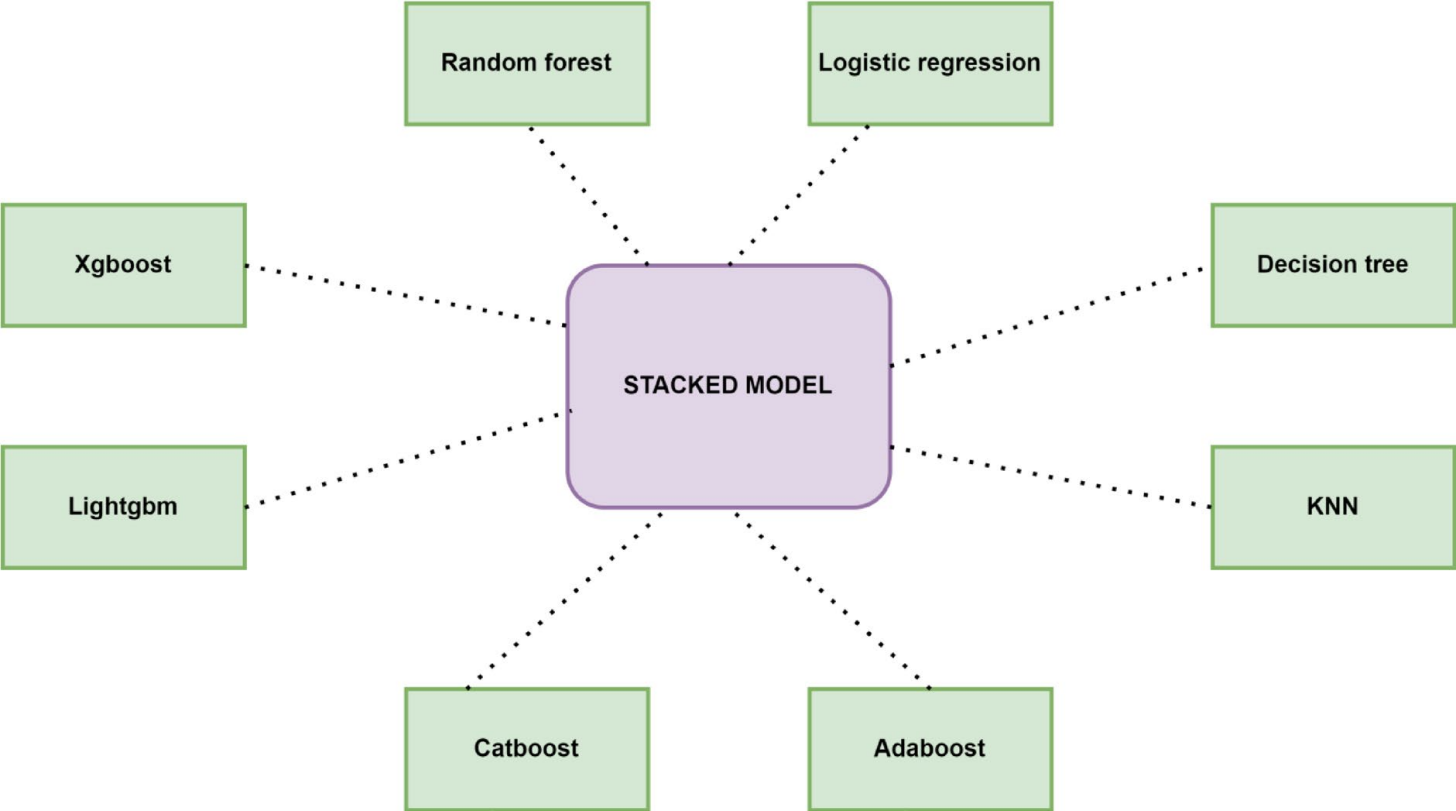
Stacking architecture.

**Fig. 8:**
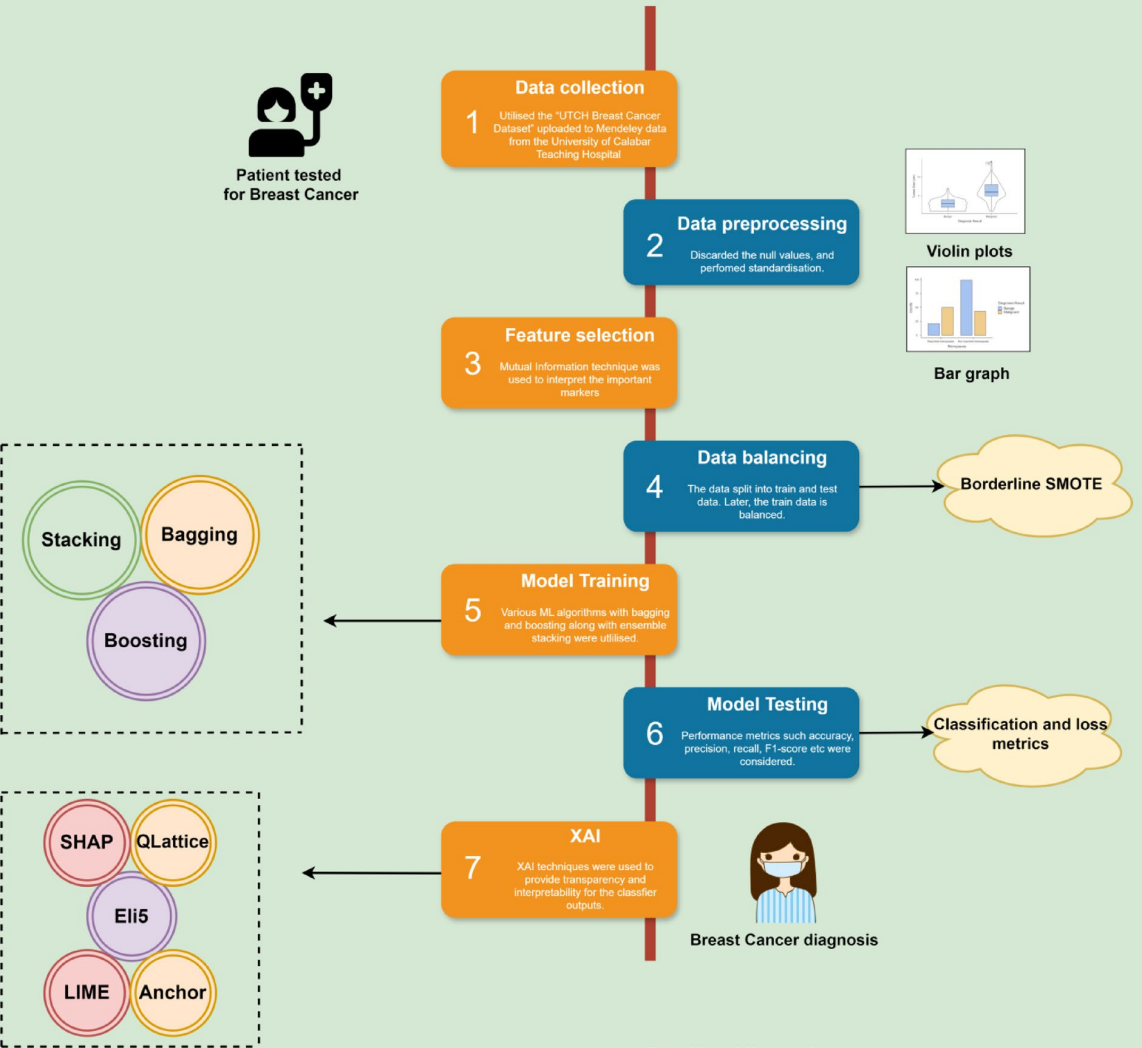
Flow diagram-based methodology.

The study employed multiple machine learning classification techniques and used a stacking algorithm to combine these classifiers. Eight classifiers used are XGBoost, LightGBM, CatBoost, AdaBoost, KNN, Decision Tree, Logistic Regression, and Random Forest. Although they use different approaches, XGBoost, LightGBM, CatBoost, AdaBoost, and Random Forest integrate multiple tree models for better performance. Decision trees, logistic regression, and K-NN work without combining multiple models. LightGBM and Xgboost are optimal for speed and performance. The outputs from the above classifiers are trained on a meta-classifier by the stacking algorithm. As the stacking methodology integrates the unique strengths from each base model, which improves the model performance by reducing the over-fitting. It also enhances the generalization as it is trained on predictions from the base models, which mitigate biases and errors that are prominent in any single model. The architecture for it is shown in Fig. 7. Hyperparameters are set before training to control how the model learns. It is performed to determine the ideal set of hyperparameters that optimize the model’s performance, generalizing the learning process to respond well to unseen data. GridSearchCV is used for hyperparameter tuning using 5-fold cross-validation in this research. A detailed flowchart of the methodology followed is shown in Fig. 8.

XAI techniques improve the model performance, interpret the results, and provide transparency to the model’s predictions. The necessity for XAI: improve the model’s reliability by identifying the cause for misclassifications; provide transparency to the model, which helps doctors in decision making and for patients to understand the rationale behind the treatment. It also helps identify the important features for the detection of breast cancer. This study used five XAI techniques: SHAP, LIME, Eli5, QLattice, and Anchor. SHAP (Shapley Additive exPlanations) is a method for interpreting complex machine learning models that assigns an important value to each feature known as the Shapley value. These values measure the impact of each input attribute on the model’s predictions^20^. It provides detailed, individualized explanations for both doctors and patients. The individual feature contribution is critical in refining the model by pinpointing unexpected feature interactions. SHAP is a model-agnostic that can be applied to any machine learning model, in this case, on STACK, a combination of various models. LIME (Local Interpretable Model-agnostic Explanations) is an agnostic model that explains the sample’s local surroundings^21^. It modifies minor aspects of data to observe the impact of the changes on the predictions, therefore facilitating the identification of the most relevant features. It is especially useful for scenarios involving specific patient predictions. It provides transparency at the individual level. LIME provides flexibility across different models without modification like SHAP. LIME provides explanations that are easy for patients or non-technical stakeholders to understand. Eli5(Explain Like I’m Five) allows us to explain the weights and predictions of the classifiers. It offers global and local explanations^22^. By analyzing the contributions of different features to the model’s predictions, Eli5 can help identify biases that might have been inadvertently introduced during the model training process. It helps understand the model’s inner workings and troubleshoot the existing issues. For instance, if a model disproportionately weighs a non-relevant feature, it might indicate overfitting or data quality issues. It is a user-friendly interface and an excellent choice for text data, like in this study, as it can explain how input text elements affect the model’s predictions, offering insights into the workings of complex models. QLattice explainability is a technique that looks for patterns and connections in data^23^. It investigates a broad range of alternative models that offer understandable explanations for the relationships in the data rather than merely fitting the data to a predetermined model. The QLattice model is a set of mathematical expressions that can connect output and input via infinite spatial paths^23^. Unlike many machine learning models, QLattice focuses on finding simple, interpretable formulas for users to understand how inputs are transformed into outputs, making the models inherently transparent. It identifies key features and provides information on how they interact with each other to affect the outcome. It is robust to new data, as it can adapt by exploring new models, making it responsive to changes in data patterns. Anchor is also a model-agnostic interpretation created by Marco Tulio Ribeiro. It makes use of if-then rules known as anchors^24^. In medical diagnosis, an anchor might specify that if certain symptoms and test results are present, the diagnosis will invariably be a specific condition. It explains a certain decision by identifying the critical factors that significantly impact it. It accurately describes why a particular decision was made, even if the overall model behavior is complex and non-linear, making it locally faithful. This method helps the user develop confidence in the model by outlining the justification for each prediction. The summary of characteristics of the XAI techniques used is shown in Table 6. Various techniques enhance the reliability and versatility of interpretative outputs through cross-verification of explanations. The study’s XAI techniques complement each other in terms of speed, adaptability, and ease of interpretability for doctors and patients. Due to the different insights in the model’s decision-making process, integrating all five techniques leverages unique strengths.

**Table 6:** Characteristics of five XAI techniques used.

XAI Technique	Characteristics
SHAP	SHAP is preferred as it is not computationally intensive on small datasets. It is model agnostic. It provides both local and global interpretability^25^
LIME	LIME is preferred as it is not computationally intensive on small datasets. It is model agnostic. It provides local interpretability. Easy to interpret for technical and non-technical stakeholders. Allows quick adaptability to new data. It is an excellent choice for text data, as in the study
Eli5	It is typically fast compared to other models. Easy to interpret for technical and non-technical stakeholders. It provides both local and global interpretability. It is an excellent choice for text data, as in the study
QLattice	It can quickly adapt to new data and discover new model relationships. It provides both local and global interpretability. It doesn’t identify bias, unlike other techniques
Anchor	Easy to interpret for technical and non-technical stakeholders. It provides coverage values to trust the specific rule. It provides local interpretability. No feature selection is involved

## Findings

### Evaluation of the model

**Fig. 9:**
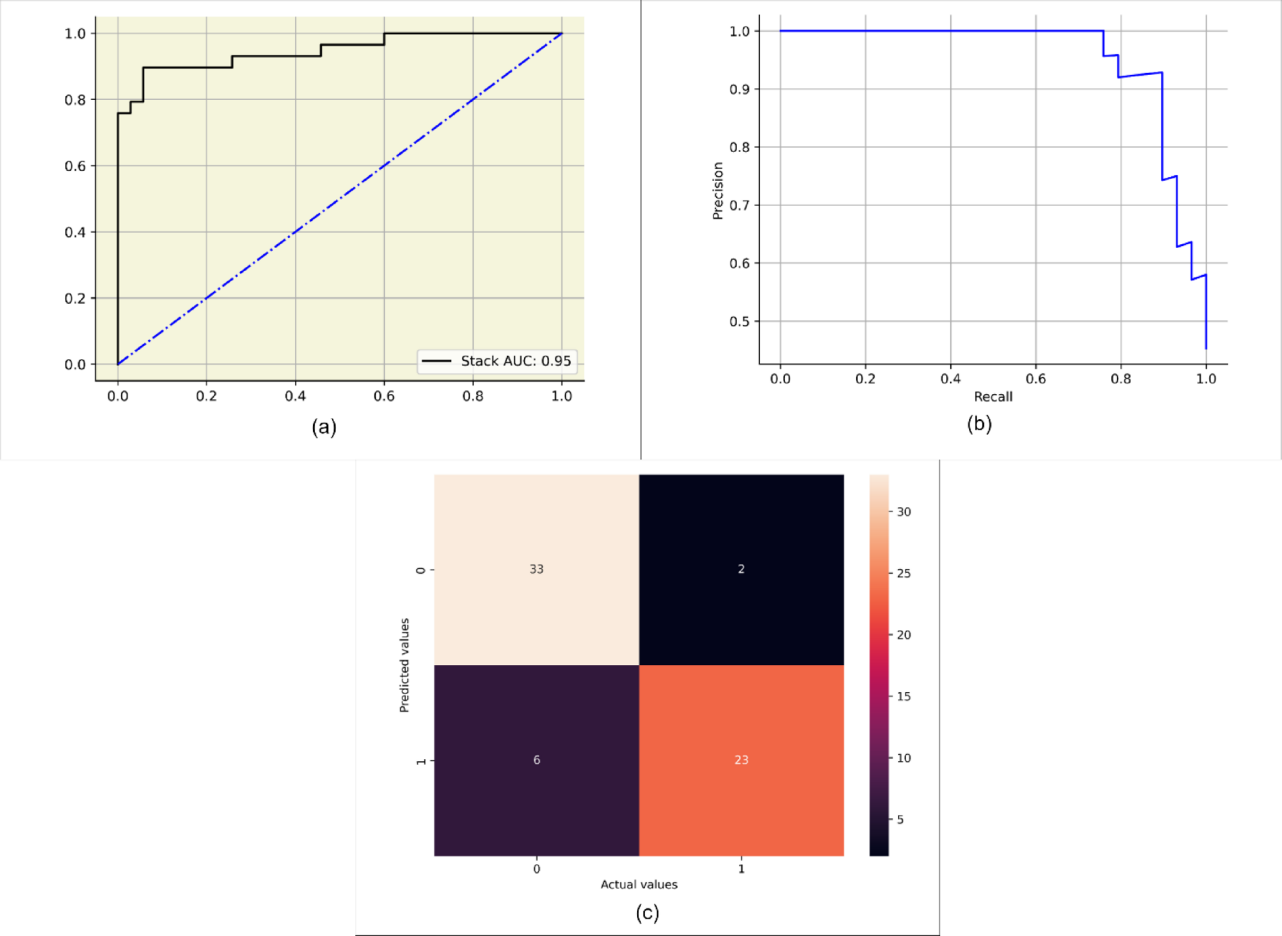
Performance of stacking classifier (**a**) AUC curve (**b**) precision-recall curve (**c**) Confusion matrix.

The thesis made use of different classifiers to predict breast cancer in patients. The algorithms were also optimized using ensemble stacking. Various evaluation metrics used are the Jaccard score, F1-score, log loss, Matthew’s correlation, accuracy, average precision, AUC, precision, recall, and Hamming loss as considering these metrics would provide a deeper insight into model evaluation. The performance of the classifiers to the mentioned metrics is shown in Table 7. Out of all the classifiers, random forest, logistic regression, and catboost gave the best performance with an accuracy of 89%. Logistic regression gave the highest precision at 87%, indicating fewer false positives than other models. Random forest, catboost, lightgbm had a recall of 84% indicating these models identify the highest proportion of true positives. Random forest with a maximum F1-score of 84%, showed a good balance between precision and recall. All the algorithms showed positive results in AUC with random forest at 0.96 with the best ability to distinguish between classes. The average precision of 0.94 was achieved by logistic regression and catboost showing good measure of precision-recall balance. The stacking classifier provided consistent and reliable results, though it does not outperform the top models like Random Forest, Logistic Regression, or Catboost. The performance of ensemble stack is shown in Fig. 9 in terms of AUC, average precision and confusion matrix which indicated the false predictions were very less resulting in a better classification model. Table 8 shows the hyperparameters obtained from each algorithm via grid search technique.

**Table 7:** Performance analysis of the machine learning classifiers using different metrics.

Algorithm	Accuracy (%)	Precision (%)	Recall (%)	F1-score (%)	Area under curve	Average precision	Hamming loss	Jaccard score	Log loss	Mathew’s correlation coefficient
Random Forest	**89**	84	**93**	**84**	**0.96**	0.93	0.11	**0.79**	**3.94**	**0.78**
Logistic Regression	**89**	**87**	90	83	0.94	**0.94**	0.11	**0.79**	**3.94**	**0.78**
Decision Tree	80	71	**93**	79	0.92	0.9	0.2	0.68	7.32	0.63
KNN	78	76	76	83	0.78	0.8	0.22	0.61	7.88	0.56
Adaboost	86	81	90	81	0.93	0.9	0.14	0.74	5.06	0.72
Catboost	**89**	84	**93**	80	0.95	0.91	**0.1**	**0.79**	**3.94**	**0.78**
Lightgbm	83	76	90	80	0.93	0.91	0.17	0.7	6.2	0.67
Xgboost	86	79	**93**	81	0.94	0.92	0.14	0.75	5.07	0.73
STACK	84	81	86	83	0.93	0.92	0.16	0.71	5.63	0.69

**Table 8:** Fine-tuning model parameters via grid Search.

Algorithm	Hyperparameter values chosen
Random forest	{‘bootstrap’: Enabled,
	‘maximum_depth’: 80,
	‘maximum_features’: 2,
	‘minimum_samples_leaf’: 3,
	‘minimum_samples_split’: 8,
	‘n_estimators’: 200}
Logistic regression	{‘C’: 1000, ‘penalty’: ‘l2’}
Decision tree	{‘criterion’: ‘gini’,
	‘maximum_depth’: 90,
	‘maximum_features’: ‘log2’,
	‘minimum_samples_leaf’: 5,
	‘minimum_samples_split’: 10,
	‘splitter’: ‘best’}
KNN	{‘no_of_neighbors’: 1}
Adaboost	{‘learning_rate’: 0.1, ‘n_estimators’: 1000}
Catboost	{‘border_count’: 5,
	‘depth’: 3,
	‘iterations’: 250,
	‘l2_leaf_reg’: 1,
	‘learning_rate’: 0.03}
Lightgbm	{‘lambda_l1’: 0,
	‘lambda_l2’: 1,
	‘minimum_data_in_leaf’: 30,
	‘num_leaves’: 31,
	‘reg_alpha’: 0.1}
Xgboost	{‘colsample_bytree’: 0.3,
	‘gamma’: 0.0,
	‘learning_rate’: 0.15,
	‘maximum_depth’: 6,
	‘minimum_child_weight’: 1}
STACK	{ use_probas = Enabled,
	average_probas = Disabled,
	meta_classifier = Logistic regression}

### Explainable AI analysis

**Fig. 10:**
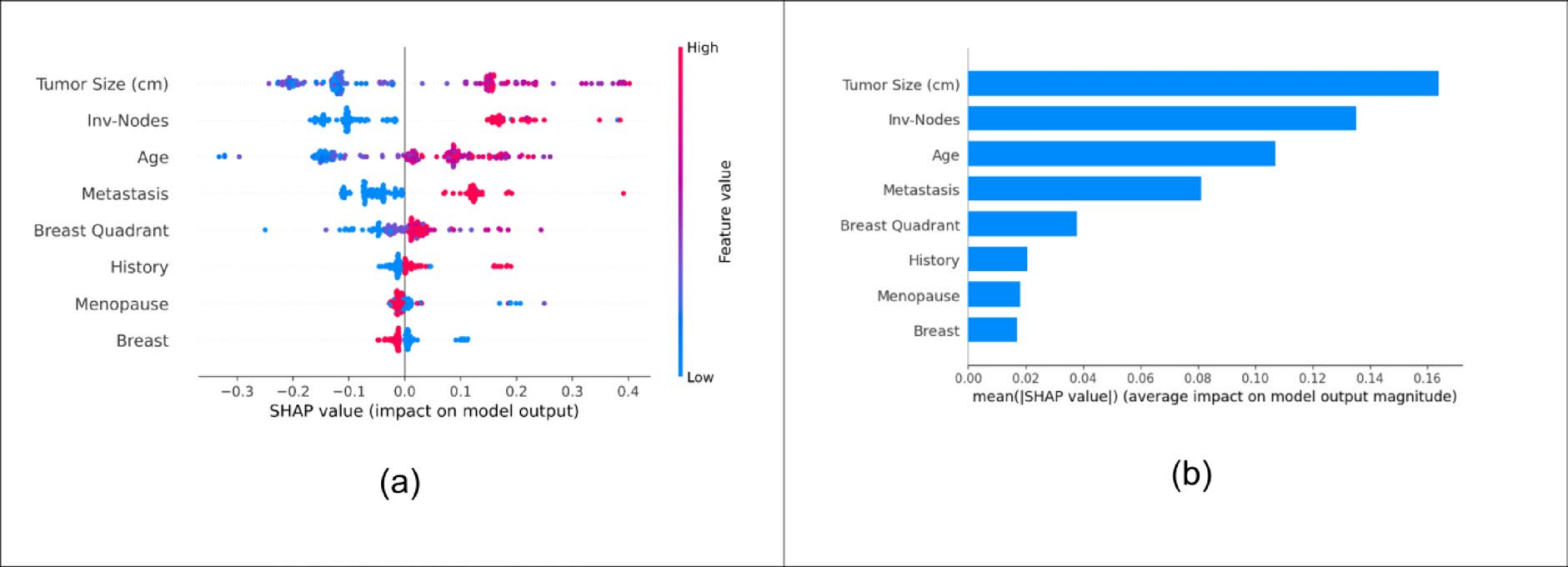
SHAP visualization through (**a**) Beeswarm plot (**b**) Mean bar plot.

The machine learning predictions were interpreted using XAI techniques to provide transparency to the various stakeholders. This study utilized five XAI techniques: LIME, Anchor, SHAP, QLattice, and Eli5. The stack model was used to interpret the results since the results are based on the ensemble of various classifiers. The SHAP (Shapley Additive Explanations) plots in Fig. 10 assist in understanding the impact of various characteristics on the prediction output of a machine learning model for breast cancer diagnosis. The beeswarm plot displays the distribution and impact of SHAP values for each feature across all instances in the dataset. The mean bar plot provides a summary of the average impact of each feature across the entire dataset. Shapley values are derived from game theory to fairly distribute the “payout” (prediction output in this context) among the “players” (features). The hyperplane at SHAP value zero separates classes 0 and 1. The right side of the line is considered malignant (class 1), whereas the left side is considered benign (class 0). The Y-axis shows the features. The feature values are color-coded from blue to red as low to high values, respectively. Each dot represents the SHAP value for a single prediction for a given feature. The color provides the feature value for the given dot. For instance, in the menopause feature, numerous cases with low menopause values are towards the side of higher SHAP value. This indicates that patients who haven’t reached menopause are likely to have benign tumors. The spread of the dots horizontally shows the variance in the impact each feature has on the model’s predictions. The mean bar plot simplifies the beeswarm plot by collapsing the individual SHAP values into a single mean absolute value per feature. Each bar represents the average impact of a feature on the model predictions, with longer bars indicating greater importance^26^. The features with less impact on the model’s prediction are breast quadrant, history, menopause, and breast. In summary, a malignant tumor is observed in cases of larger tumor size, involved nodes, old age, and metastasis. SHAP interpretability gives the explainability for a certain patient as well the overall model. By providing a clear ranking of these features, SHAP supports risk assessment by pointing out key areas for clinical focus, potentially guiding biopsy or imaging decisions.

**Fig. 11:**
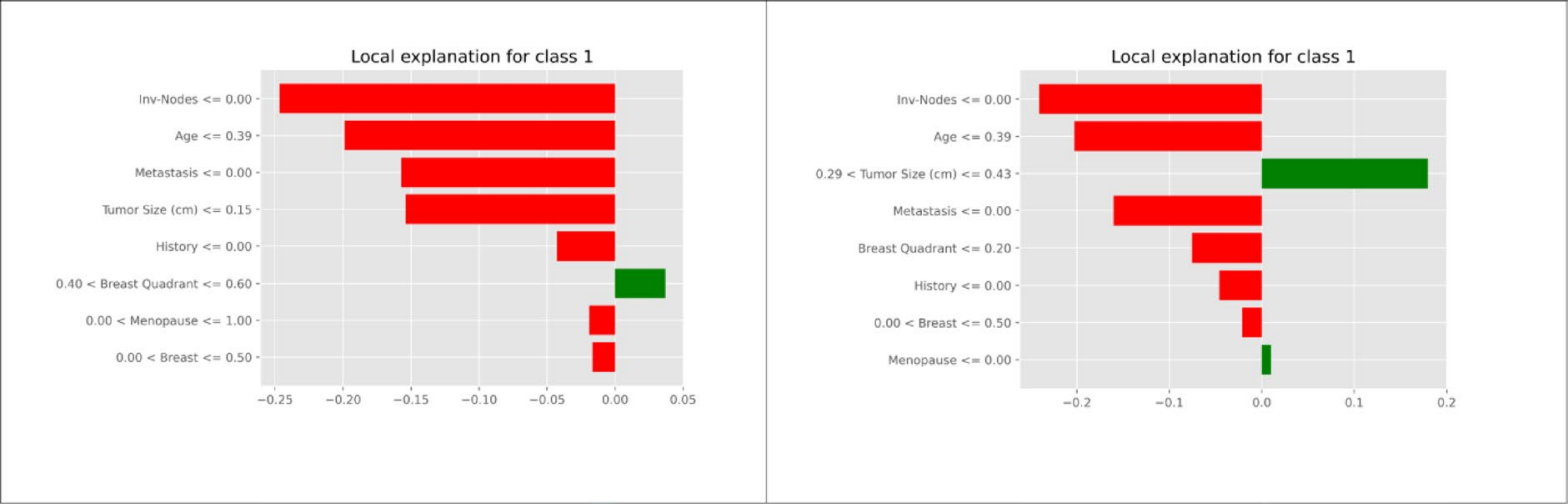
LIME analysis for different patients.

LIME plots provide a granular view of how each feature affects the prediction for an individual instance. It approximates the complex model locally with a simpler, interpretable model. The surrogate model in LIME is used to generate these explanations by approximating around specific instances. LIME generates new samples around the instance being explained by slightly altering its features. The feature importances derived from this simple model serve as the explanation. Features with larger absolute weights are more important for predicting the specific instance. The local explanation for class 1(malignant) instance is shown in Fig. 11. The X-axis represents prediction probability. Each bar in the plot represents a feature. Positive values mean that the feature’s state contributes positively to the model’s prediction for class 1. Negative values mean that the feature’s state negatively influences the prediction for class 1. For example, the tumor size < = 0 is a bar towards the negative value, which indicates the particular feature contributes to the model in less likely predicting it as class 1. The features are arranged in the order of their contribution. From LIME, it is evident that the involved nodes, age, metastasis, and tumor size are the important features. LIME evaluation can help analyze individual patient data as to how each feature contributed to a particular diagnosis. LIME can assist a clinician comprehend how minor changes in features might modify the diagnosis, allowing for a more nuanced understanding that may affect follow-up advice or monitoring frequency.

**Fig. 12:**
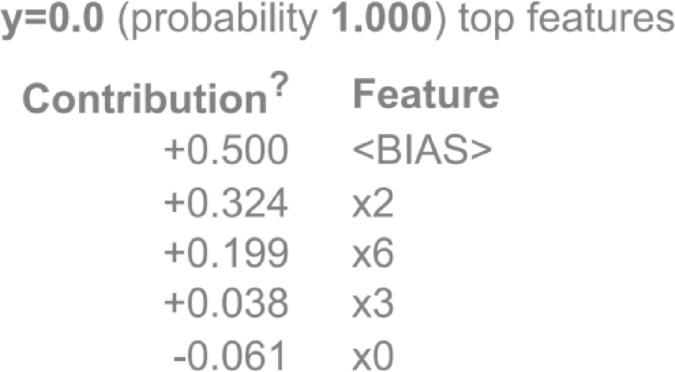
Eli5 interpretation (x0 - age, x2 - tumor size, x3 - inv-nodes and x6 - breast quadrant).

ELI5 also provides insights into the model’s general logic by aggregating the contributions of features across a large set of predictions. In Fig. 12, each row represents the contribution of a feature to the model’s prediction. These are listed as x0, x2, x3, x6, and a bias term. x0 as age, x2 as tumor size, x3 as inv-nodes and x6 as breast quadrant. Next to each feature is the bias value contributing to the model prediction. A positive number means it has a higher contribution in predicting output, while a negative number suggests it has a low contribution in predicting output. The tumor size is + 0.324, which provides strong evidence for prediction. According to Eli5, age, tumor size, inv-nodes, and breast quadrant are important features. By reviewing the global importance of features, clinicians can evaluate which attributes significantly influence the model across various scenarios, helping to identify potential biases or points of failure. This allows practitioners to test the model’s logic against their clinical experience, making model outputs easier to accept and integrate into routine diagnostic processes.

**Fig. 13:**
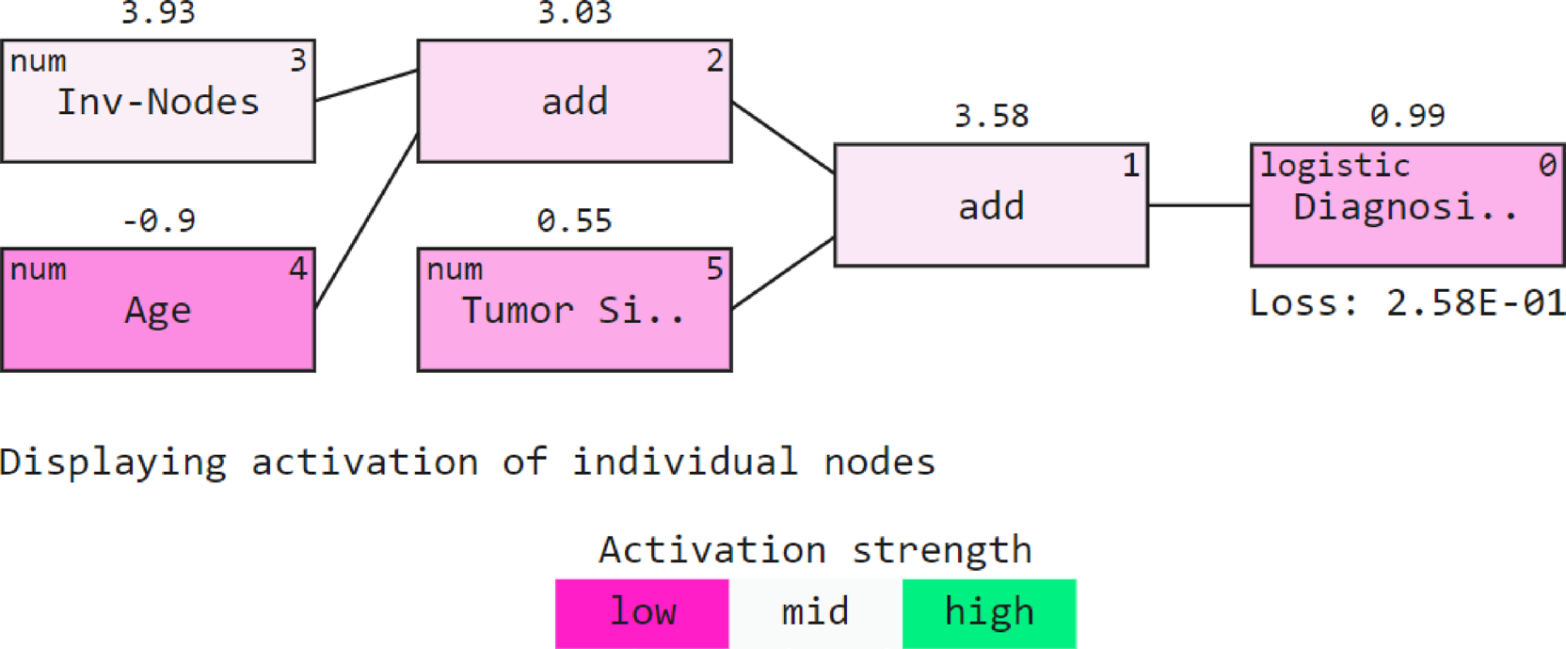
QGraph analysis.

Figure 13. shows a visualization of machine learning model analysis using QLattice which explores model architectures and understands feature interactions. QGraph represents a potential mathematical model or equation that describes how inputs (features) in the dataset are related to the output (target). It consists of nodes and edges, where each node represents a mathematical operation (like addition, multiplication, or more complex functions), and edges represent the data flow between these operations. The structure is directed, meaning data moves from input nodes towards an output node in a specified direction. Tumor size, Inv-nodes, and age are considered important markers. The activation strength shows how strongly the node is influencing the output. In this case, all nodes seem to activate moderately, suggesting balanced contributions to the model’s output. The output of the final addition is then fed into a logistic function that transforms the input into a value between 0 and 1. Conclusively, the Qgraph provides a mathematical model that best fits the predictions. Unlike other XAI techniques, QLattice does not offer the ease of understanding for non-technical stakeholders. However, due to its versatility, the QLattice can adapt to new data, providing a promising interpretation. This technique complements clinical workflows by allowing doctors to quickly identify primary signs, making it easier to incorporate AI findings into traditional diagnostic processes.

Anchors are conditions with different possibilities of features that, when met, can predict an output with certainty. Table 9. displays anchor explanations, where each row shows an anchor rule, precision, and coverage. Precision is the likelihood that the prediction is the same for a given condition. Coverage states the number of cases for which the anchor is met in the dataset. When the condition is met, they predict a particular outcome accurately. Inv-Nodes > 0.00 AND Age > 0.64 indicates the prediction is malignant with the likelihood of 0.99 observed from the dataset. This provides ease of interpretation and transparency by considering the combination of feature values. This clarity supports consistent and trustworthy diagnostic choices by providing doctors with a useful checklist of high-impact criteria to take into account. All these XAI techniques complement other techniques to provide transparency and trust in the model for physicians and patients.

**Table 9:** Anchors with precision and coverage.

Anchors	Prediction	Precision	Coverage
Age < = 0.40 AND Tumor Size (cm) < = 0.29	Benign	0.97	0.23
Tumor Size (cm) < = 0.14 AND Inv-Nodes < = 0.00	Benign	0.9	0.27
Age < = 0.40 AND Tumor Size (cm) < = 0.14	Benign	1	0.12
Tumor Size (cm) < = 0.14 AND Inv-Nodes < = 0.00	Benign	0.89	0.28
Tumor Size (cm) < = 0.29 AND Inv-Nodes < = 0.00	Benign	0.87	0.55
Inv-Nodes > 0.00 AND Tumor Size (cm) > 0.29	Malignant	0.98	0.32
Inv-Nodes > 0.00 AND Age > 0.64	Malignant	0.99	0.15
Tumor Size (cm) > 0.29 AND Inv-Nodes > 0.00	Malignant	0.99	0.31
Age < = 0.53 AND Tumor Size (cm) < = 0.14	Malignant	0.99	0.21
Inv-Nodes > 0.00 AND Age > 0.53	Malignant	1	0.26

## Discussion

This study used the “UTCH Breast Cancer Dataset” taken from Mendeley data. The dataset used was sourced from the University of Calabar Teaching Hospital, Nigeria. It had 213 patient observations with nine features. We examined the performance of nine different classifiers for predicting breast cancer in patients. Five XAI approaches were also used to understand the predictive results. The findings are different among various classifiers. The metric considered majorly was the F1-score. It is crucial in medical applications since it is the harmonic average of precision and recall. Greater precision values result in fewer false positives, reducing unnecessary medical tests, treatments, and patient anxiety. Higher recall can avoid missing actual breast cancer cases, minimizing false negatives. The focus was on the F1 score, it considers the reduction of false negatives and false positives. Random forest produced optimal results, with an F1-score of 84%. The stack ensemble model was used to balance the biases and optimize the strengths of individual classifiers. It showed an F1-score performance of 83%. The false positives and false negatives observed by the stack classifier are two and six, respectively (out of 65 test cases). A comparison with radiologist reviews was done in earlier studies, where the F1 score was 84.88% on a dataset of 752 patients on numerical data^9^. While the performance of the STACK ensemble model is close to radiologist reviews, it suggests the model’s reliability in comparison to state-of-the-art diagnostic tools. The models proposed can aid the radiologist in making decisions faster with minimal human errors. In accordance with all five XAI techniques, the crucial features considered are age, inv-nodes, and tumor size in decreasing order importance. Additionally, LIME considered the breast quadrant an important marker. Higher age contributed negatively to the prediction of breast cancer. The existence of affected nodes raises the likelihood of the disease. Larger tumor sizes showed a greater risk of the illness. These XAI techniques can provide invaluable insights at different stages in a clinical workflow. SHAP and LIME could assist in initial screenings by highlighting key predictive factors, while ELI5 and QLattice could support more in-depth reviews by revealing diagnostic pathways and high-impact indicators. Anchor ensures that certain conditions are met before a diagnosis is confirmed, which provides additional support in critical decision points. These markers can be used to identify the diagnosis precisely. Further, the findings of this study are consistent with previously published medical studies^27–30^.

The research compared statistical hypothesis tests, information gain, and XAI techniques to identify important features. Table 10 streamlines the feature’s importance in all three analyses. Tumor size, age, and involved nodes were all critical factors in all approaches. Mutual information and statistical tests considered metastasis a significant feature. Chi-square tests considered menopause and breast quadrant as important markers in diagnosing breast cancer. These markers can help an ML model predict the condition of breast cancer. All three methods consider tumor size, age, and involved nodes as important breast cancer markers The comparison provides cross-validation of features considered necessary from different analyses. Statistical tests provide the importance of features by measuring the association (chi-square) or differences in means (t-test) between variables. This makes it more biased towards the dataset. It is also limited to linear relationships. Mutual information measures the amount of information one variable contains about another. Both statistical analysis and mutual information indicate important individual features; it might not fully reveal how combinations of features interact to impact the model’s prediction. XAI techniques used in this study ensure a promising effort to do the same.

**Table 10:** Important features chosen by statistical analysis, mutual information, and XAI techniques.

Features	Chi-square/t-tests	Mutual Information	XAI
Tumor size	✓	✓	✓
Age	✓	✓	✓
Menopause	✓	×	×
Involved nodes	✓	✓	✓
Breast Quadrant	✓	×	×
Metastasis	✓	✓	×
Breast	×	×	×
History	×	×	×

Many studies have incorporated the use of ML in medical diagnosis. Sharma et al.^11^ and Thilaka et al.^12^ conducted studies on implementing machine learning techniques in breast cancer diagnosis. While the same dataset was used by both studies, the difference in selecting features and classifiers impacted diagnosis. This indicates the adaptability and potential of machine learning to not only diagnose diseases but also to tailor models according to specific clinical objectives, enhancing their practical utility in real-world applications. The addition of XAI to provide transparency and interpretability for the model can help understand the model and explain the result, enabling physicians to prevent misdiagnoses. In spite of assisting clinicians in making decisions, it reassures patients regarding the rationale behind the recommended treatment. Various studies have integrated XAI with ML models to provide transparency and explainability, enhancing its utility in medical diagnosis^31^. An effort was made to develop a better model using XAI. Few studies published have used ensemble models along with XAI. Aravena et al.^13^ utilized Indonesian women’s breast cancer data, which consisted of numerical data. The research was focused on demographic-specific data. Features such as age at first pregnancy, breastfeeding duration, and high-fat diet were identified as highly impactful. Another research approached using ensemble learning to diagnose breast cancer^14^. This study used a Wisconsin dataset from UCI ML Respiratory that contained 32 features derived from images. It used SHAP to promote transparency in ML decisions. Islam et al.^15^ made use of geometric features derived from images. A total of seven features were used, including the diagnosis parameter. This study applied SHAP to elucidate the output. Unlike other studies, Imouokhome et al.^16^ focused on using cellular-level images. It targeted pixel-intensity variations for classification. It also employed ResNet50, a deep CNN architecture that was pre-trained using transfer learning in the study. XAI techniques, such as Integrated Gradient, GradientShap, and Occlusion, were applied to understand the histopathological images’ key features. Oztekin et al.^9^ evaluated the model with nine features extracted from images. When the same data was reviewed by a radiologist, the accuracy was exceptional, demonstrating the ML classifier’s reliability. The paper also interpreted models using SHAP and LIME. Table 11 presents a comparative analysis of the above studies, including our research. Even though other studies produced positive findings, they did not employ as many XAI methodologies as the present study. Further, the XAI results were further validated using mutual information and hypothesis testing. This study used statistical analysis and mutual information to identify important markers along with XAI techniques. This helps offer a thorough comprehension of feature importance, which can improve decision-making, interpretability, and model performance. While emphasizing performance, the research focused on the F1 score as a metric since the diagnosis is sensitive to false positives and false negatives. Also, the study employed GridCVSearch for hyperparameter optimization. Unlike most studies, multiple XAI techniques are accounted for in the paper. Combining the various techniques yields a dependable interpretation of the model as each has a distinct strength. It also helps bridge the gap between global and local interpretations. Some of the XAI techniques are model agnostic, while others are model-specific. This provides flexibility in making an appropriate conclusion. Moreover, the XAI techniques used are simpler and relatively straightforward so that anyone can easily apply them. Integrating a stack ensemble and five XAI techniques highlights the work of this paper as a significant contribution to breast cancer detection.

There are a few limitations in the paper. The model’s performance could be further improved using deep-learning approaches^32,33^, and the dataset’s size can be increased to reduce overfitting and handle high variance. The dataset used was purely numerical, which limits the diversity of features chosen^34^. The research focused on data from a specific demographic area (Nigeria). This could lead to a domain mismatch, i.e., the model might not perform well when tested for diversified data. Improvements for the study can be incorporated by utilizing data from diverse demographics. Validation of the model can be done on external datasets to ensure adaptability^35^. The ML models can be compared with clinical expert decisions to avoid misdiagnoses. In the future, the research can be extended by increasing the dataset size and diversity to provide generalizability. A parallel review of the test cases can be done by biopsy or radiological imaging to provide validation by real-time diagnosis. This strengthens the connection to clinical practice. Improvements can be made using raw image data to provide more accessibility in choosing features. A standardized feature set across various researchers can facilitate collaborative research, allowing for quicker advancements.

**Table 11:** Overview of similar studies.

References	Dataset size	No. of features	ML models used	Performance	XAI Techniques
^11^	569 instances	32 attributes	Three classifiers	95.90% accuracy, 98.27% precision, 94.20% F1 score	None
^12^	500 samples	5 features	Two classifiers	93% accuracy	None
^13^	400 records of Indonesian women	Not mentioned	Four classifiers	85% accuracy, 85.4% precision	SHAP
^14^	569 instances	32 features	Several classifiers with ensemble models	99.99% F1 score, 99.99% recall, 99.99% precision, 99.99% accuracy	SHAP
^15^	500 records of Bangladeshi patients	7 features	Five classifiers	96% F1 score, 95% recall, 94% precision, 97% accuracy	SHAP
^16^	7907 histopathological images	No numerical features	ResNet50 CNN model	96.84% accuracy	Integrated Gradient, GradientShap, Occlusion
^9^	752 patients	11 features	Six classifiers along with radiologist reviews	94.34% accuracy, 92.11% precision, 93.96% F1 score	SHAP and LIME
This research	213 patients	9 features	Nine classifiers + Custom Stack ensemble model	84% F1 score	**SHAP**,** LIME**,** Eli5**,** Qlattice and Anchor**

## Conclusion

Breast cancer is a condition of abnormal growth of cancer cells in the breast tissue. If untreated, it can spread to the surrounding tissues with a fatality risk. This study used machine learning approaches and explainable AI to predict breast cancer detection. Multiple machine-learning algorithms were utilized to determine whether the tumor was benign or malignant. The stack ensemble model presented in this paper is better in generalization and robustness than individual models. It is also insensitive to bias. Various XAI techniques were used to provide the black box explainability to the model’s output. Rather than highly computational techniques, the focus was utilizing novel methods that were easy to understand even for medical personnel. The results obtained using XAI techniques were validated using mutual information and statistical hypothesis techniques. The classifiers can be incorporated in hospitals, diagnostic centers, pathological labs, and research trials. Utilizing these models can provide an early diagnosis and hence reduce breast cancer fatalities.

## Data Availability

Data was obtained from the Mendeley data repository. https://data.mendeley.com/datasets/63fpbc9cm4/2.
